# New records of the genus *Iporhogas* Granger (Hymenoptera, Braconidae, Rogadinae) from Vietnam, with description of four new species

**DOI:** 10.3897/zookeys.428.7729

**Published:** 2014-07-24

**Authors:** Khuat Dang Long

**Affiliations:** 1 Institute of Ecology & Biological Resources (IEBR), Vietnam Academy of Science & Technology (VAST), 18 Hoang Quoc Viet Road, Cau Giay, Ha Noi, Vietnam

**Keywords:** Braconidae, Rogadinae, *Iporhogas*, new record, new species, Vietnam

## Abstract

The genus *Iporhogas* Granger, 1949 (Braconidae: Rogadinae) is recorded for the first time for Vietnam. Four new species of the genus *Iporhogas*, viz. *Iporhogas albilateralis* **sp. n.**, *I. contrastus* **sp. n.**, *I. simulatus* **sp. n.** and *I. tricoloratus* **sp. n.**, from Vietnam are described and illustrated, and additionally, one species, *Iporhogas guangxiensis* Chen & He, 1997, is newly recorded for Vietnam’s fauna of the family Braconidae. A key to the five Vietnamese species of the genus *Iporhogas* and a checklist with distributions of the ten species are provided.

## Introduction

The Rogadinae is one of the largest subfamilies of Braconidae, as far as known species of this subfamily consists of koinobiont endoparasitoids of lepidopteran larvae in which the host caterpillar is mummified (van Achterberg 1991; Townsend and Shaw 2009). Little is known of the subfamily Rogadinae from Vietnam, and since 2007, some papers on Vietnamese Rogadinae with description of 17 new species have been published (Long and van Achterberg 2007, 2008a, 2008b). To date only seven genera and 25 species of Vietnamese Rogadinae have been reported in papers scattered in the literature (Long and Belokobylskij 2003; Long and van Achterberg 2008a, 2008b; Butcher et al. 2012).

The genus *Iporhogas* proposed by Granger (1949) is a small genus of the rogadine braconids, this genus comprises six species, of which five occur in the Oriental region (Chen and He 1997) and one in the Afrotropical region (Granger 1949). Six *Iporhogas* species were keyed by Chen and He (1997) with five species described and illustrated as new from China, but without any information on their hosts.

For several years the author has been collecting Braconidae from all over Vietnam to gain an understanding of the braconid fauna of Vietnam. In this paper four new and aberrant species of *Iporhogas* from Vietnam present in the Braconidae Collection of Vietnam National Museum of Nature (VNMN) are described and illustrated. A checklist and the distribution of ten species of the genus *Iporhogas* are given, in addition one species is recorded for the first time from Vietnam, together they represent the first record of the genus *Iporhogas* for Vietnam.

## Material and methods

Specimens studied are deposited in the Collection of the Institute of Ecology & Biological Resources (IEBR) and Vietnam National Museum of Nature (VNMN) at HaNoi, assembled by the author during numerous expeditions in Vietnam. The specimens of *Iporhogas* were mainly collected by using sweep nets or malaise traps set in open habitats as secondary or impoverished forests.

All the types are kept in Vietnam National Museum of Nature (VNMN) (HaNoi, Vietnam), but two females designated as paratypes of *Iporhogas albilateralis* sp. n. (‘Rog.282’) and *Iporhogas contrastus* sp. n. (‘Rog.367’), and one male designated as paratype of *Iporhogas tricoloratus* sp. n. (‘Rog.363’) are donated to Naturalis Biodiversity Center (RMNH), Leiden, The Netherlands.

Terminology used in this paper follows van Achterberg (1993), sculpture terms are based on Harris (1979), and vein terminology follows the modified Comstock-Needham system (van Achterberg 1993). For diagnosis of the Rogadinae see van Achterberg (1991); for identification and subdivision of the subfamily, see van Achterberg (1993); for a key to the genera of the subfamily Rogadinae, see Chen and He (1997); for additional references and data, see Yu et al. (2013). The photographs were taken with a Canon G15 camera attached to an Olympus SZ61 binocular microscope at IEBR. Abbreviations used in this paper are as follows: POL = postocellar line; OOL = ocular-ocellar line; Od = diameter of posterior ocellus; MT: Malaise trap; ‘Rog. + number’: code number indexing for specimens of the Rogadinae in the collection; N: North; S: South, NC: North Central, NE: Northeast, NW: Northwest; NP: National Park; NR: Nature Reserve.

## Results

### Checklist and distribution of *Iporhogas* species

*Iporhogas albilateralis* sp. n., from Vietnam

*Iporhogas contrastus* sp. n., from Vietnam

*Iporhogas chinensis* Chen & He, 1997, from China

*Iporhogas flavistigma* Chen & He, 1997, from China

*Iporhogas guangxiensis* Chen & He, 1997, from China, Vietnam

*Iporhogas infuscatipennis* Granger, 1949 from Madagascar

*Iporhogas rugivertex* Chen & He, 1997, from China

*Iporhogas simulatus* sp. n., from Vietnam

*Iporhogas tricoloratus* sp. n., from Vietnam

*Iporhogas unicolor* Chen & He, 1997, from China

## Systematics

### Iporhogas

Taxon classificationAnimaliaHymenopteraBraconidae

Granger, 1949

**Figures 1–4.**
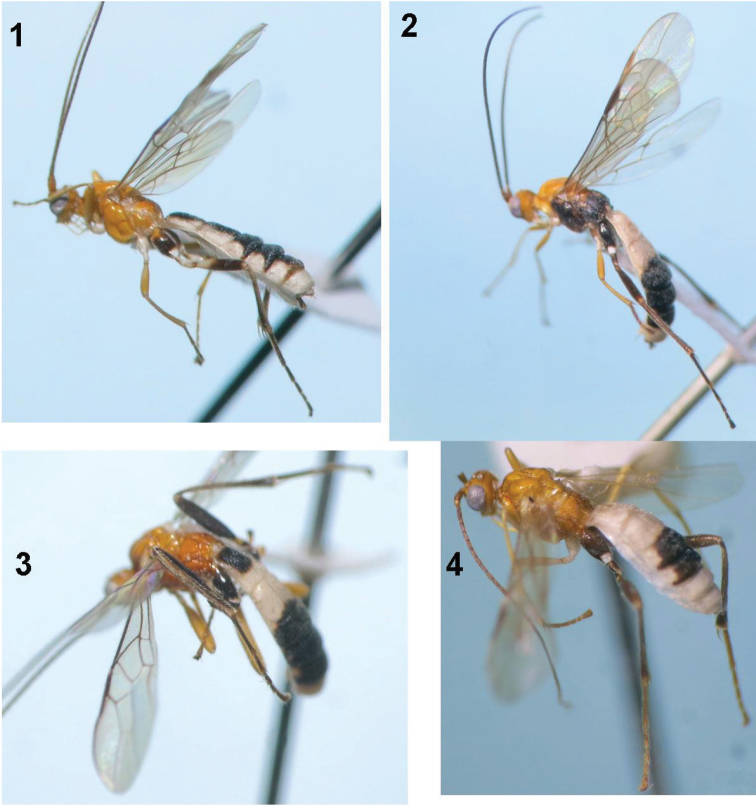
**1** *Iporhogas albilateralis* sp. n., female habitus **2** *Iporhogas contrastus* sp. n., female habitus **3** *Iporhogas tricoloratus* sp. n., female habitus **4** *Iporhogas tricoloratus* sp. n., male habitus.

Figs 1 [Fig F2] [Fig F3] [Fig F4] [Fig F5] –34

Iporhogas Granger, 1949: 167. Type-species (by monotypy): *Iporhogas infuscatipennis* Granger, 1949.

#### Diagnosis.

**Figures 5–10. F2:**
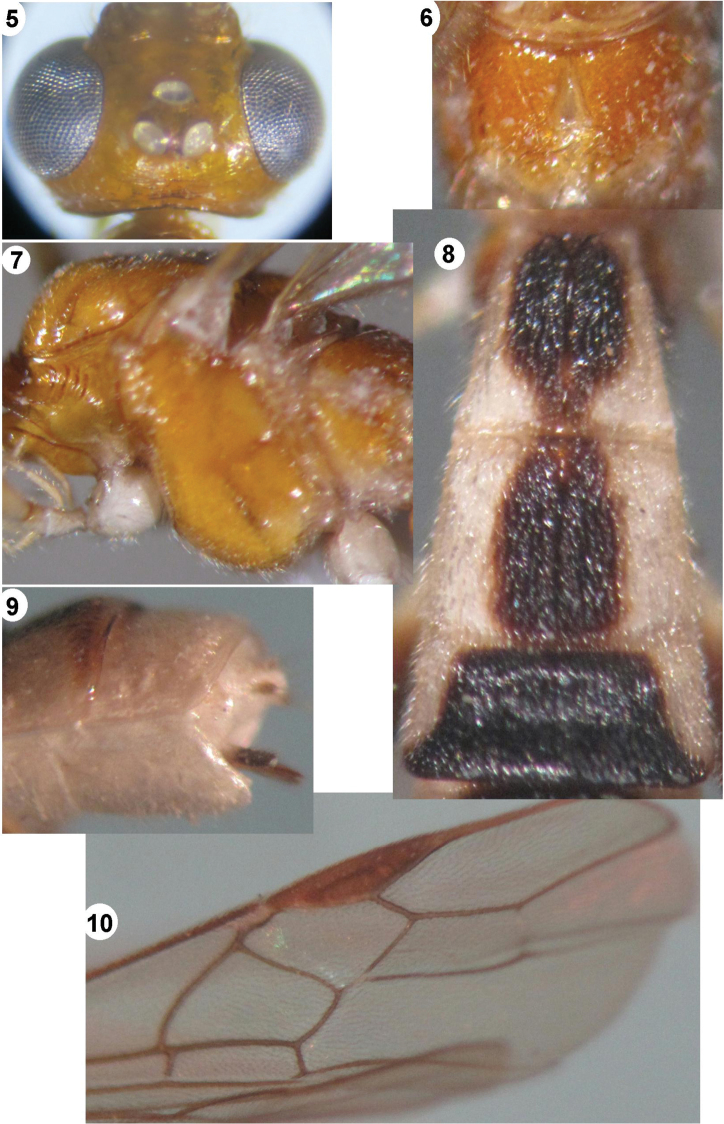
*Iporhogas albilateralis* sp. n., female. **5** head dorsal **6** propodeum dorsal **7** mesosoma lateral **8** metasomal tergites 1-3 dorsal **9** apex of metasoma lateral **10** fore wing.

**Figures 11–16. F3:**
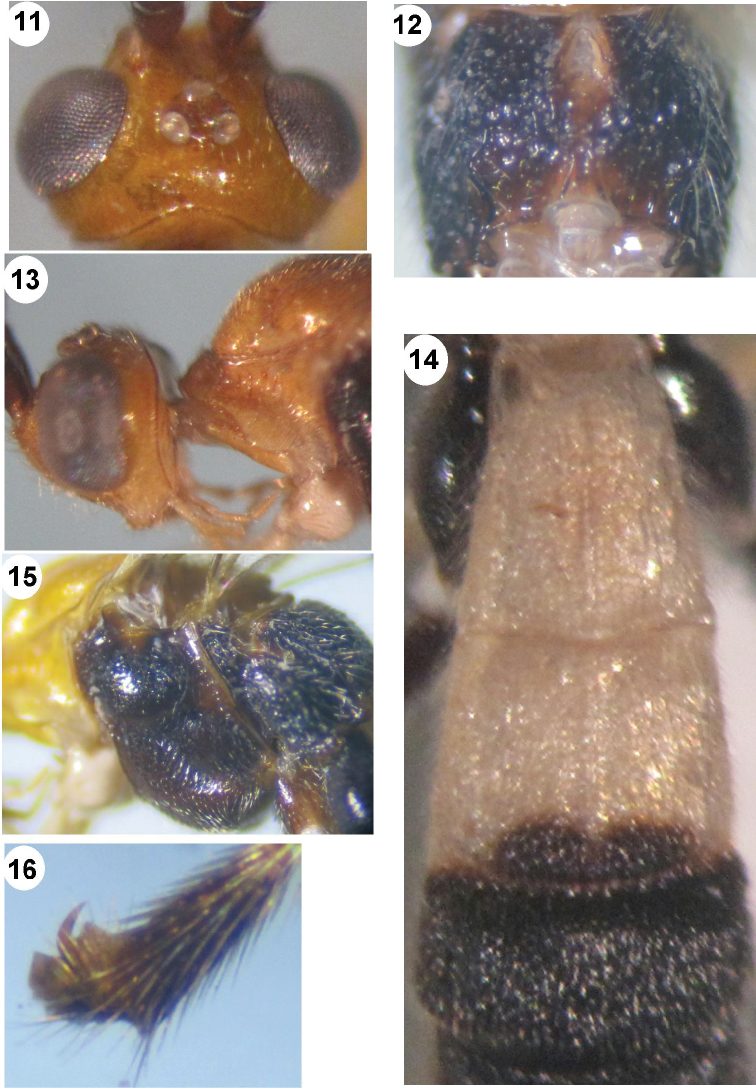
*Iporhogas contrastus* sp. n., female. **11** head dorsal **12** propodeum dorsal **13** head lateral and propleuron **14** metasomal tergites 1-3 dorsal **15** mesosoma lateral **16** hind tarsal claw lateral.

**Figures 17–23. F4:**
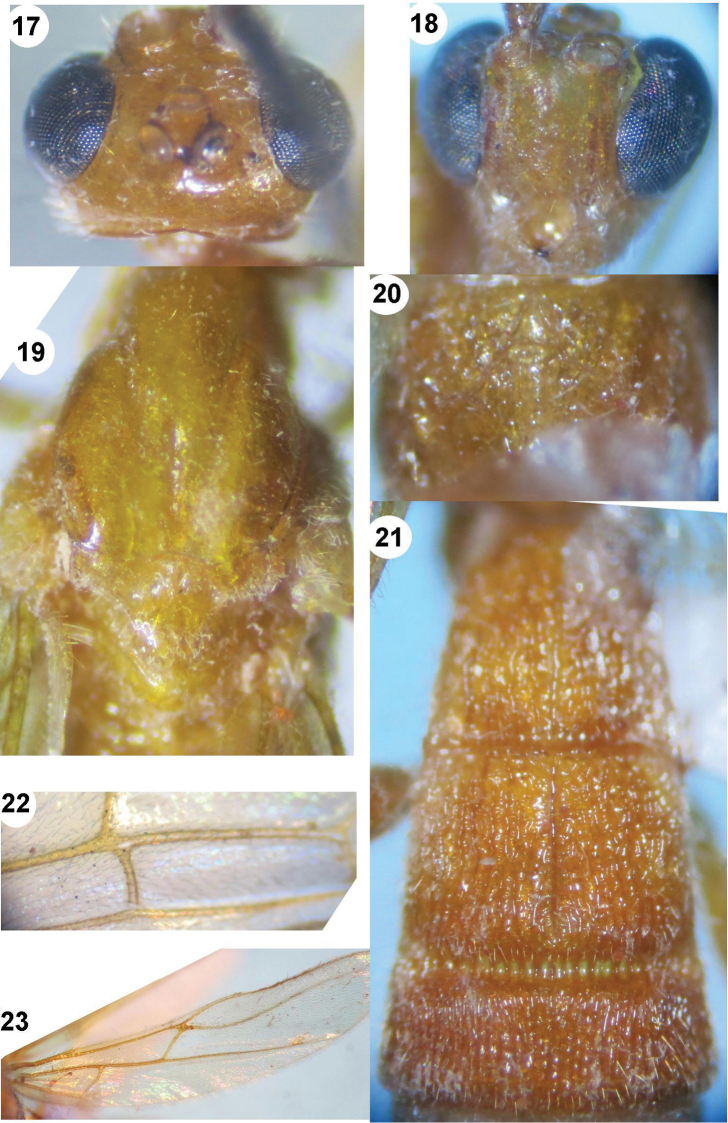
*Iporhogas simulatus* sp. n., female. **17** head dorsal **18** head frontal **19** mesonotum **20** propodeum dorsal **21** metasomal tergites 1–3 dorsal **22** veins 1-CU1 and cu-a of fore wing **23** hind wing.

**Figures 24–28. F5:**
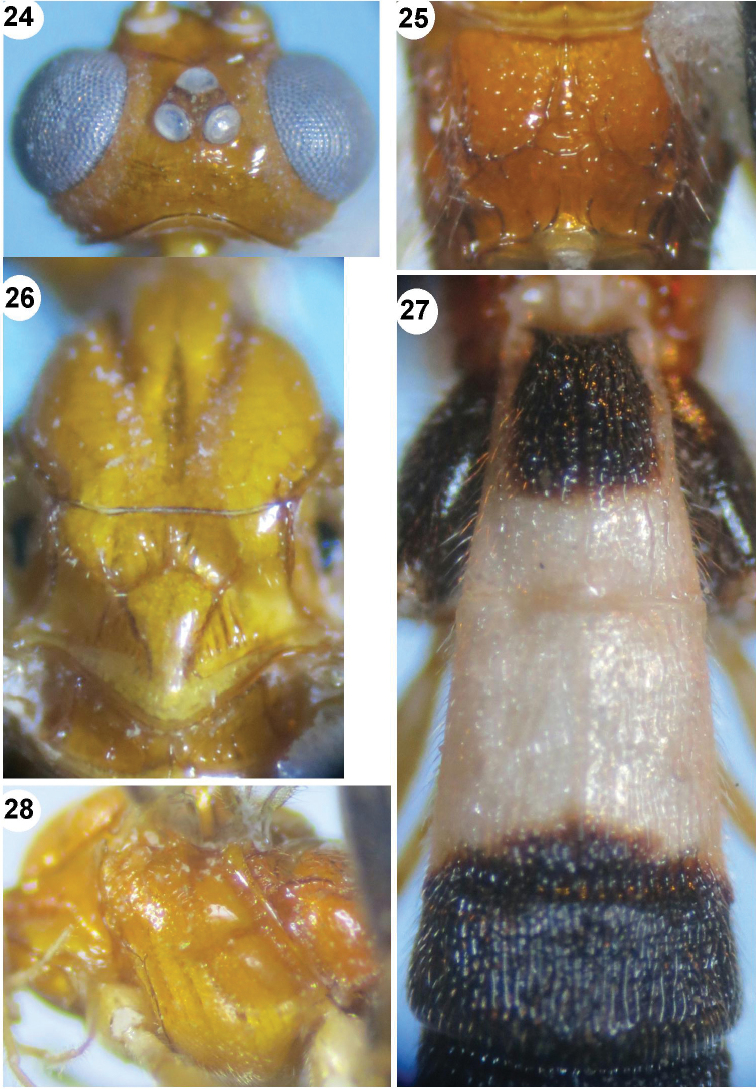
*Iporhogas tricoloratus* sp. n., female. **24** head dorsal **25** propodeum dorsal **26** mesonotum **27** metasomal tergites 1–3 dorsal **28** mesosoma.

**Figures 29–34.**
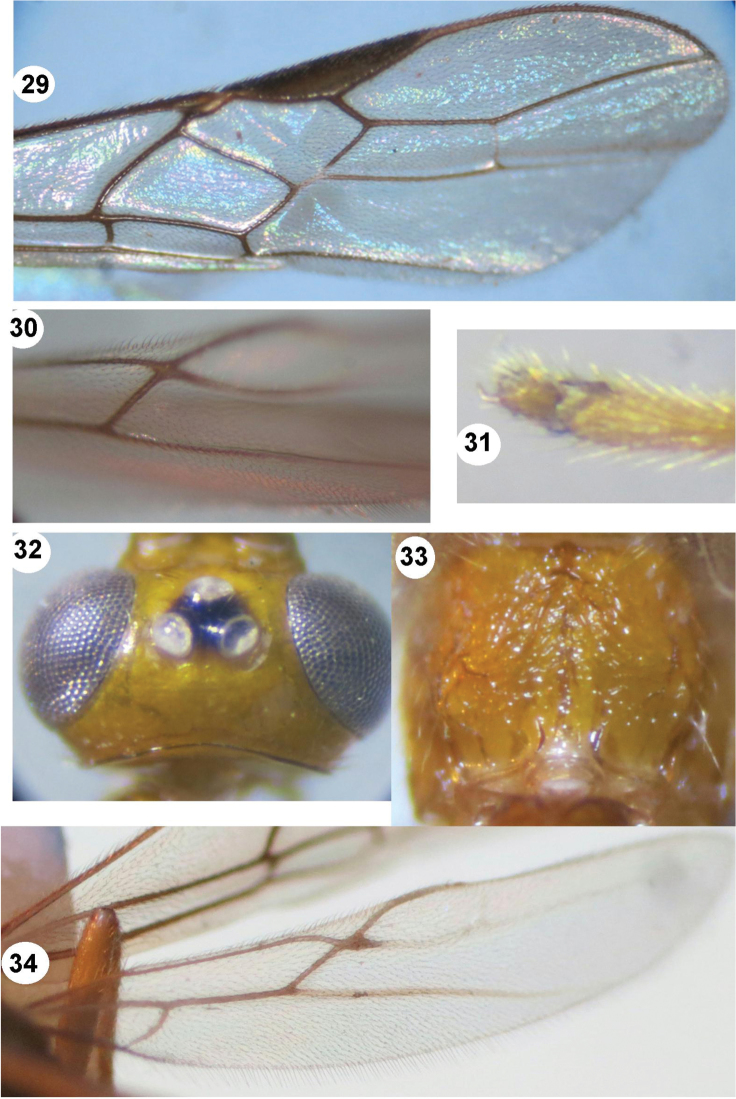
**29–30** *Iporhogas tricoloratus* sp. n., female. **28** mesosoma lateral **29** fore wing **30** hind wing (in part) **31–34** *Iporhogas guangxiensis* Chen & He, 1997, female. **31** hind tarsal claw lateral **32** head dorsal **33** propodeum dorsal **34** hind wing.

Antennal segments 47–54 (female), 36–43 (male), apical segment with spine; maxillary and labial palpi of female normal; hypostomal carina joining occipital carina ventrally (Fig. 13); occipital carina complete and concave (Figs 5, 11, 17, 24, 32); vertex rugose or transversely rugose; frons rugose; malar suture shallow; eyes emarginate (Fig. 18); precoxal sulcus shallow and narrow, absent anteriorly and posteriorly; mesopleuron smooth or finely and sparsely punctate; notauli rather wide and crenulate; vein 1-SR of fore wing medium-sized, continuous with vein 1-M (Figs 10, 29); vein m-cu of fore wing antefurcal, curved, gradually merging into vein 2-CU1, and converging to vein 1-M posteriorly; vein 3-SR of fore wing longer than 2-SR (Figs 10, 29); vein 1-CU1 short; vein 1-SR+M of fore wing sinuate; vein cu-a of fore wing nearly vertical; vein M+CU1 of fore wing nearly straight; vein 1-M of hind wing straight; vein 1r-m of hind wing comparatively short and oblique (Figs 23, 34); vein m-cu of hind wing absent; tarsal claws single or with large rounded lobe; hind tibial spurs curved, glabrous or setose basally; apex of hind tibia with distinct comb of specialized setae at inner side; propodeum areolate because of (partly) developed submedial carinae (Figs 6, 12, 20, 25, 33); propodeal tubercles absent; first metasomal tergite with large dorsope (Figs 14, 21, 27), its dorsal carinae united behind level of spiracles and without basal flanges; second tergite with comparatively large medio-basal triangular area, connected to a medio-longitudinal carina (Figs 8, 14, 21, 27); fourth-fifth tergites with sharp lateral crease (Fig. 9); hypopygium of female medium-sized to large; ovipositor sheath rather slender (Fig. 9).

#### Key to species of the genus *Iporhogas* from Vietnam

**Table:** 

1	Tarsal claws simple, without lobe (Fig. 31)	2
–	Tarsal claws with large acute lobe (Fig. 16)	3
2	Occipital carina in dorsal view angularly concave (Fig. 17); propodeum with small triangular areola and without basal carina (Fig. 20); vein 1-SC+R of hind wing almost straight apically (Fig. 23); vein 2-SC+R of hind wing quadrate (Fig. 23)	*Iporhogas simulatus* sp. n.
–	Occipital carina in dorsal view roundly concave (Fig. 32); propodeum without areola and with basal carina; vein 1-SC+R of hind wing distinctly curved apically (Fig. 34); vein 2-SC+R of hind wing subquadrate, swollen apically (Fig. 34)	*Iporhogas guangxiensis* Chen & He
3	Occipital carina in dorsal view slightly concave (Fig. 5); vein 1-CU1 of fore wing nearly quadrate (Fig. 10); two basal metasomal tergites black medially (Fig. 8)	*Iporhogas albilateralis* sp. n.
–	Occipital carina in dorsal view deeply concave (Figs 11, 24); vein 1-CU1 of fore wing transverse (Fig. 29); first metasomal tergite white or black basally; second tergite black apically (Figs 14, 27)	4
4	Propodeum reddish yellow (Fig. 25); precoxal sulcus narrow and punctate (Fig. 28); ocelli rather large, distance between anterior and posterior ocelli 0.4 times as long as diameter of ocellus (Fig. 24)	*Iporhogas tricoloratus* sp. n.
–	Propodeum black (Figs 12, 15); precoxal sulcus wide and distinctly crenulate (Fig. 15); ocelli smaller, distance between anterior and posterior ocelli equal to diameter of ocellus (Fig. 11)	*Iporhogas contrastus* sp. n.

### Descriptions

#### Iporhogas albilateralis sp. n.

Taxon classificationAnimaliaHymenopteraBraconidae

http://zoobank.org/3D5BE644-6BBA-4884-B392-BC0433A51531

Figs 1 , 5–10

##### Material.

Holotype, female (VNMN), ‘Rog.608’, “[NE Vietnam:] Vinh Phuc, Tam Dao NP, 110m, bushes, 06.x.2008, KD Long”. Paratypes; 1 female (VNMN), ‘Rog.224’, “[S Vietnam:] Kien Giang, Phu Quoc island, garden, 21.vii.2002, KD Long”; 1 female (RMNH), ‘Rog.282’, “ [NW Vietnam:] Hoa Binh, Yen Thuy, fruit orchard, MT, 01-10.ix.2002, KD Long”.

##### Description.

Holotype, female, body length 6.1 mm, fore wing length 4.6 mm (Fig. 1).

*Head.* Antenna broken, with 35 segments remaining; middle segments 3.0 times longer than wide (6:2); third antennal segment 1.1 times fourth segment (9:8); width of face equal to length of face and clypeus combined; malar space 1.2 times as long as mandible width (6:5); mandible width 0.7 times as long as hypoclypeal depression (5:7); in dorsal view height of eye 3.2 times as long as temple (16:5); occipital carina weakly concave (Fig. 5); in lateral view, width of eye 3.0 times as long as temple (15:5); ocelli large, POL:Od:OOL=2:5:4; distance between anterior and posterior ocelli 0.75 times as long as OOL (3:4) (Fig. 5); face rugose-punctate with circular fine striae around antennal socket; frons shiny, finely rugose; temple smooth; vertex shiny with fine curved striae (Fig. 5).

*Mesosoma.* Length of mesosoma 1.45 times as long as high (70:48); propleuron wide and deep, crenulated anteriorly (Fig. 7); mesoscutum shiny with sparse fine punctures; notauli deep, crenulated anteriorly, smooth posteriorly; scutellar sulcus 0.4 times as long as scutellum (5:12) with one medial carina; scutellum shiny, smooth; precoxal sulcus shallow, punctate; mesopleuron shiny, largely smooth medially (Fig. 7), anterior area of mesopleuron and mesosternum with sparse fine punctures; metapleuron punctate; propodeum areolate medially, punctate basally, rugose apically (Fig. 6).

*Wings.* Fore wing: pterostigma 3.8 times as long as wide (38:10); r:2-SR:3-SR:SR1=7:13:20:34; vein r arising from middle of pterostigma; vein 1-SR+M distinctly S-shaped; vein cu-a vertical, postfurcal; vein 1-CU1 near subquadrate (Fig. 10), 1-CU1:cu-a:2-CU1 =2:7:23; basal length of second submarginal 2.5 times as long as its apical width (32:13). Hind wing: vein M+CU 1.2 times as long as vein 1-M; M+CU:1-M: 1r-m=28:24:13; vein 2-SC+R transverse.

*Legs.* Hind coxa shiny, with oblique fine striae dorso-apically; length of hind femur, tibia and basitarsus 4.8, 9.0 and 9.0 times as long as their width, respectively; inner hind tibial spur 0.4 times as long as basitarsus (14:36); hind tarsal claw with large lobe.

*Metasoma.* First tergite 1.1 times as long as its apical width (33:31) (Fig. 8); medially second tergite 1.5 times longer than third tergite (30:20); second suture wide, crenulate; first and second tergites with medial longitudinal carina; third-fifth tergites with dense parallel striae on whole surface; ovipositor sheath 0.75 times as long as hind basitarsus (9:12); ovipositor sheath straight (Fig. 9).

*Colour.* Body bicoloured; scapus yellow; antennal segments brownish yellow; palpi pale yellow; fore and middle legs yellow; hind coxa black, yellow basally; hind trochanter and trochantellus white; hind femur black, white basally; hind tibia yellowish brown basally, darkish apically; hind tarsus brown; pterostigma and vein yellowish brown; propodeum brownish yellow; first-second metasomal tergites black medially, white laterally; third-fifth tergites largely black medially, white baso-laterally; sixth tergite white entirely; sternites ivory.

##### Variation.

Paratypes with 54 antennal segments; first tergite 1.1–1.25 times as long as apical width; medial length of metasomal second tergite 1.5 times as long as third tergite medially; body length 6.2–6.5 mm; fore wing length 4.7–4.8 mm.

##### Male.

Unknown.

##### Distribution.

NE Vietnam: Vinh Phuc; NW Vietnam: Hoa Binh; S Vietnam: Phu Quoc Island.

##### Biology.

Unknown.

##### Etymology.

From “albus” (Latin for “white”) and “lateralis” (Latin for “of the side”), because of the white lateral side of the metasoma.

##### Notes.

*Iporhogas albilateralis* sp. n. can be distinguished from other species by having the hind coxa with oblique striae dorso-apically. The new species differs from *Iporhogas chinensis* Chen & He, 1997, from China by having the precoxal sulcus shallow, punctate; mesopleuron finely and sparsely punctate; metapleuron punctate and vein cu-a of fore wing distinctly postfurcal. The new species also differs from *Iporhogas flavistigma* Chen & He, 1997, from China by having the occipital carina in dorsal view roundly concave; precoxal sulcus shallow, sparsely punctate; metapleuron largely punctate and vein M+CU of hind wing slightly longer vein 1-M.

#### Iporhogas contrastus sp. n.

Taxon classificationAnimaliaHymenopteraBraconidae

http://zoobank.org/F166F73A-585D-4931-9152-BE14743ACC6C

Figs 2 , 11–16

##### Material.

Holotype, female (VNMN), ‘Rog.713’, “[NW Vietnam:] Hoa Binh, Mai Chau, Tan Son, orchard, MT, 20°43'10.3"N, 104°59'47.0"E, 650m, 1–5.v.2010, KD Long”. Paratypes: 1 female (RMNH), ‘Rog.367’, “[NW Vietnam:] Lai Chau, Tam Duong, Lai Nhi Thang, forest, 09.x.2004, KD Long”; 1 female (VNMN), ‘Rog.047’, “[NC Vietnam:] Ha Tinh, Huong Son, Rao An, forest, 200m, 22.v.1998, KD Long”.

##### Description.

Holotype, female, body length 6.1 mm, fore wing length 5.0 mm (Fig. 2).

*Head.* Antenna broken, with 51 segments remaining; middle segments twice longer than wide (6:3); third antennal segment 1.3 times fourth segment (9:7); width of face 1.1 times length of face and clypeus combined (19:17); malar space 0.85 times as long as mandible width (6:7); mandible width 0.9 times as long as hypoclypeal depression (7:8); in dorsal view height of eye 2.8 times as long as temple (17:6); occipital carina deeply concave (Fig. 11); in lateral view width of eye 3.0 times as long as temple (15:5); POL:Od:OOL=3:4:4; distance between anterior and posterior ocelli equal to OOL (Fig. 11); face with short median carina, rugose medially, lateral area with fine transverse striae; frons with fine transverse striae; vertex and temple smooth.

*Mesosoma.* Length of mesosoma 1.6 times as long as high (72:45); propleuron wide, crenulated (Fig. 13); notauli deep, crenulate; mesonotum with sparse fine punctures; scutellar sulcus 0.5 times as long as scutellum (6:12) with one medial carina; scutellum sparsely punctate; mesopleuron shiny and smooth medially, sparsely and finely punctate anteriorly and posteriorly; precoxal sulcus wide, crenulate (Fig. 15); mesosternum sparsely punctate; metapleuron rugose-punctate; propodeum areolate medially, rugose-punctate anteriorly, rugose latero-posteriorly (Fig. 12).

*Wings.* Fore wing: pterostigma 4.4 times as long as wide (44:10); r:2-SR:3-SR:SR1=10:13:26:42; vein r arising before middle of pterostigma; vein 1-SR+M nearly straight; vein cu-a distinctly postfurcal; 1-CU1:cu-a:2-CU1=3:6:28; basal length of second submarginal 2.5 times as long as its apical width (35:14). Hind wing: vein M+CU 1.3 times vein; M+CU:1-M:1r-m=32:24:12; vein 2-SC+R transverse.

*Legs.* Hind coxa sparsely punctate dorsally; length of hind femur, tibia and basitarsus 5.7, 8.75 and 9.0 times as long as their width, respectively; inner hind tibial spur 0.33 times as long as basitarsus (12:36); hind tarsal claw with large acute lobe (Fig. 16).

*Metasoma.* First tergite 1.2 times as long as its apical width (40:33) (Fig. 14); first-second tergites sparsely striate; medial length of second tergite 1.5 times third tergite (30:20); first-second metasomal tergites with medial longitudinal carina, sparsely striate (Fig. 14); third tergite without medial carina; third-fifth tergites densely striate; ovipositor sheath as long as hind inner spur; ovipositor slender.

*Colour.* Scapus and antennal segments brown; palpi yellow; stemmaticum black; pronotum and mesonotum yellow; mesopleuron, metapleuron and propodeum black; fore coxa ivory; fore femur, tibia and tarsus yellow, except telotarsus brownish yellow; middle coxa brown, fore femur, tibia and tarsus yellow, except telotarsus brownish yellow; hind coxa black, hind femur, tibia and tarsus blackish brown; fore wing yellowish brown; pterostigma yellow with brown border; veins yellowish brown; first and second tergites white, except black spot at apex of second tergite; third-fifth tergites black; tergite 6 white; hypopygium ivory with long sparse setae; ovipositor sheath brown.

##### Variation.

Paratypes: antenna with 49 segments; first tergite 1.2 times as long as its apical width; medial length of metasomal second tergite 1.3–1.5 times third tergite medially; body length 6.1–7.8 mm; fore wing length 5.5–6.6 mm; stemmaticum brown; lateral lobes of mesoscutum dark brown; first metasomal tergite with brown spot at base.

##### Male.

Unknown.

##### Distribution.

NW Vietnam: Hoa Binh (Mai Chau), Lai Chau (Tam Duong); NC Vietnam: Ha Tinh (Huong Son).

##### Biology.

Unknown.

##### Etymology.

From “contra” (Latin for “opposite”), because of the contrasting orange and black of its body colour.

##### Notes.

*Iporhogas contrastus* sp. n. differs from *Iporhogas chinensis* Chen & He, 1997, from China by having: the metapleuron rugose-punctate; precoxal sulcus wide, crenulate; hind tarsal claw with large lobe; vein cu-a of fore wing distinctly postfurcal; vein M+CU of hind wing 1.3 times as long as vein 1-M and hind leg entirely black. The new species differs from *Iporhogas flavistigma* Chen & He, 1997, from China by having: the occipital carina in dorsal view deeply concave; precoxal sulcus wide, crenulate; metapleuron largely punctate; vein 2-SC+R of hind wing transverse and hind leg entirely black.

#### Iporhogas simulatus sp. n.

Taxon classificationAnimaliaHymenopteraBraconidae

http://zoobank.org/EB11CDBE-E11A-4B2F-9A05-AD0B1DFC2DA7

Figs 17–23

##### Material.

Holotype, female (VNMN), ‘Rog.798’, “[C Vietnam:] Thua Thien-Hue, A Luoi, A Roang, forest, 700m, 28.v.2006, HV Tru”.

##### Description.

Holotype, female, body length 7.5 mm, fore wing length 5.8 mm.

*Head.* Antenna broken, with 19 segments remaining; middle segments 1.75 times longer than wide (7:4); third antennal segment 1.4 times fourth (10:7); width of face 1.1 times length of face and clypeus combined (22:20); malar space 0.8 times as long as mandible width (7:9); mandible rugose, mandible width 0.9 times as long as hypoclypeal depression (9:10); malar suture present; in dorsal view height of eye 2.85 times as long as temple (20:7), occipital carina in dorsal view angularly concave (Fig. 17); in lateral view width of eye 2.25 times as long as temple (18:8); ocelli large, POL:Od:OOL=3:7:5; distance between anterior and posterior ocelli 0.8 as long as OOL (4:5) (Fig. 17); face transversely rugose (Fig. 18); frons transversely rugose; vertex and temple smooth.

*Mesosoma.* Length of mesosoma 1.5 times as long as high (98:66); propleuron wide and deep, crenulate; mesoscutum almost smooth, with sparse fine punctures; notauli deep and crenulate anteriorly, flat posteriorly; mesoscutum with deep smooth medial depression between notauli (Fig. 19); scutellar sulcus 0.5 times as long as scutellum (8:15); scutellum with sparse fine punctures; precoxal sulcus wide and shallow, sparsely rugose-punctate; mesopleuron and mesosternum smooth; metapleuron sparsely rugose; propodeum with small triangular areola and without basal carina, sparsely rugose laterally (Fig. 20).

*Wings.* Fore wing: pterostigma 4.1 times as long as wide (53:13); r:2-SR:3-SR:SR1=12:20:31:58; vein r arising from middle of pterostigma; vein 1-SR+M slightly curved; vein cu-a slightly postfurcal; vein 1-CU1 very short and nearly quadrate (Fig. 22); cu-a:2-CU1=9:31; basal length of second submarginal 3.6 times as long as its apical width (47:13). Hind wing: vein M+CU 1.3 times as long as vein 1-M (39:29); M+CU:1-M: 1r-m=39:29:16; vein 2-SC+R quadrate (Fig. 23).

*Legs.* Hind coxa largely smooth dorsally, sparsely and finely punctate ventrally; length of hind femur, tibia and basitarsus 5.3, 8.8 and 9.0 times as long as their width, respectively; inner hind tibial spur 0.3 times as long as basitarsus (14:45); hind tarsal claw simple.

*Metasoma.* First tergite 1.1 times as long as its apical width (47:44) (Fig. 21); medial length of second tergite 1.9 times third tergite (36:24); first-second tergites with medial longitudinal carina; ovipositor sheath 0.6 times as long as hind basitarsus (9:14).

*Colour.* Yellow; scapus and pedicel yellow; eyes and antennal segments brown; palpi yellow; first-third metasomal tergites yellow, fourth-sixth tergites pale yellow; wing vein yellow; pterostigma yellow, brownish apically.

##### Male.

Unknown.

##### Distribution.

NC Vietnam: Thua Thien-Hue (A Luoi, A Roang).

##### Biology.

Unknown.

##### Etymology.

Named from “simulo” (Latin for “imitate, copy”), because this species is similar to *Iporhogas guangxiensis* Chen & He, 1997.

#### Iporhogas tricoloratus sp. n.

Taxon classificationAnimaliaHymenopteraBraconidae

http://zoobank.org/FA89EC6F-FD71-4530-AFF0-3CF4DCBE1591

Figs 3 , 4 , 24 –30

##### Material.

Holotype, female (VNMN), ‘Rog.629’, “[NC Vietnam:] Quang Binh, Xuan Trach, Phong Nha-Ke Bang NP, forest, sweeping net, 18.iv.2010, KD Long”. Paratypes: 10 males, ‘Rog.053’ (VNMN), “[NW Vietnam:] Hoa Binh, Yen Thuy, forest, 20°28'12N, 105°34'40E 80m, 4.v.2002”; ‘Rog.259’ (VNMN), “[NW Vietnam:] Hoa Binh, Yen Thuy, forest, MT 20°23'06N, 105°34'11E 300m, 10–20.vii.2002; KD Long”; ‘Rog.360, Rog.361, Rog.362’ (VNMN), ‘Rog.363’ (RMNH), “[NW Vietnam:] Son La, Moc Chau, Xuan Nha NR, forest, 11.x.2004, KD Long”; ‘Rog.495’ (VNMN), “[NE Vietnam:] Ha Giang, Vi Xuyen, Cao Bo, forest, 300m, 11.v.2007, KD Long”; ‘Rog.555’ (VNMN), “[NE Vietnam:] Ha Giang, Vi Xuyen, Ngoc Duong, bushes, 18.x.2006, KD Long”; ‘Rog.598’ (VNMN), “[NE Vietnam:] Vinh Phuc, Tam Dao NP, 200m, 05.ix.2008, KD Long”; ‘Rog.633’ (VNMN), “[NW Vietnam:] Phu Tho, Xuan Son, MT 21°14'N, 104°57'E 140m, 1–5.x.2009, KD Long, NH Thao”.

##### Description.

Holotype, female, body length 6.3 mm, fore wing length 5.0 mm (Fig. 3).

*Head.* Antenna with 47 segments; middle segments 2.8 times longer than wide (7.0:2.5); third segment 1.1 times fourth (8:7); width of face as long as length of face and clypeus combined; malar space 0.7 times as long as mandible width (5:7); mandible width 0.9 times as long as hypoclypeal depression (7:8); malar suture present; in dorsal view, height of eye 3.4 times as long as temple (17:5), occipital carina roundly concave (Fig. 24); in lateral view, width of eye 4.2 times as long as temple (25:6); POL:Od:OOL=2:5:3.5; distance between anterior and posterior ocelli 0.6 times as long as OOL (2:3.5) (Fig. 24); face rugose medially, transversely striate laterally; frons smooth with divergent striae close to antennal sockets; vertex with fine transverse striae; temple smooth.

*Mesosoma.* Length of mesosoma 1.5 times as long as high (73:50); propleuron deep and crenulate anteriorly, shallower posteriorly and finely crenulate; mesoscutum shiny with deep groove medially (Fig. 26), finely sparsely punctate; notauli narrow, deep and crenulate anteriorly, flat and smooth posteriorly; scutellar sulcus 0.6 times as long as scutellum (6:10), with 3 carinae (Fig. 26); scutellum almost smooth; precoxal sulcus short and shallow, crenulate (Fig. 28); mesopleuron sparsely and finely punctate anteriorly, smooth posteriorly; mesosternum sparsely punctate; metapleuron finely punctate; propodeum areolate medially, depressed posteriorly, sparsely punctate anteriorly, almost smooth posteriorly (Fig. 25).

*Wings.* Fore wing: pterostigma 4.6 times as long as wide (32:7); r:2-SR:3-SR:SR1=7:11:18:45; vein r arising near middle of pterostigma; vein 1-SR+M weakly S-shaped; vein cu-a distinctly postfurcal (Fig. 29), 1-CU1:cu-a:2-CU1:3-CU1=2:4:21:4; basal length of second submarginal 3.2 times as long as its apical width (35:11). Hind wing: vein M+CU 1.4 times as long as 1-M; M+CU:1-M:1r-m=35:25:13; vein 2-SC+R subquadrate (Fig. 30).

*Legs.* Upper side of hind coxa shiny and with sparse fine punctures; length of hind femur, tibia and basitarsus 4.8, 8.4 and 9.0 times as long as their width, respectively; inner hind tibial spur 0.4 times as long as basitarsus (11:30); hind tarsal claw with large lobe.

*Metasoma.* First tergite 1.3 times as long as its apical width (37:28) (Fig. 27); first-second tergites with medial carinae and sparse longitudinal striae, third-fifth tergites without medial carina and densely striate, sixth tergite sparsely striate; medial length of second tergite 1.6 times than third (33:21); second suture wide, crenulate; hypopygium with long rather dense setae; ovipositor sheath as long as hind basitarsus.

*Colour.* Tricoloured body; scapus and pedicellus yellowish brown; antennal segments brown; palpi pale yellow; eyes brown; mesosoma yellow; fore and middle legs yellow; hind leg black, except coxa, trochanters and trochantellus black; hind tibia and tarsus brown; pterostigma and wing veins yellowish brown; first metasomal tergite black medio-basally, white apically and baso-laterally; second tergite white, except black apex; third-fifth tergites black entirely; sixth tergite entirely white; hypopygium white; ovipositor sheath 0.7 times as long as hind inner spur (8:11).

##### Variation.

Paratypes (males; Fig. 4), antenna with 36–43 segments; first tergite 1.1–1.3 times as long as its apical width; medially second metasomal tergite 1.3–1.5 times as long as third tergite medially; body length 4.5–5.2 mm; fore wing length 3.6–4.4 mm.

Mesopleuron of males sometimes with fine transverse crenulae anteriorly; mesopleuron rugose-punctate anteriorly; fore and middle legs pale yellow; hind coxa and femur brown; hind tibia and tarsus yellowish brown; pterostigma and wing veins of males yellow or yellowish brown; first and second metasomal tergites white or ivory, except black spot at base of first and at apex of second tergite; third-fourth tergites black, except white basal corners; fifth and sixth tergites entirely white.

##### Distribution.

NW Vietnam: Lai Chau (Tam Duong), Hoa Binh (Yen Thuy), Phu Tho (Xuan Son); NE Vietnam: Ha Giang (Vi Xuyen), Vinh Phuc (Tam Dao NP); NC Vietnam: Quang Binh (Phong Nha-Ke Bang NP).

##### Biology.

Unknown.

##### Etymology.

From “tri” (Latin for “three”), and “coloris” (Latin for “hue, tint”), because of the tricoloured body.

##### Notes.

*Iporhogas tricoloratus* sp. n. differs from *Iporhogas chinensis* Chen & He, 1997, from China by having: the ocelli large; metapleuron finely punctate and vein M+CU of hind wing comparatively long, 1.4 times as long as vein 1-M. The new species differs from *Iporhogas flavistigma* Chen & He, 1997, from China by having the occipital carina in dorsal view roundly concave; metapleuron finely punctate and hind coxa finely punctate.

#### Iporhogas guangxiensis

Taxon classificationAnimaliaHymenopteraBraconidae

Chen & He, 1997

Figs 31–34

##### Material.

1 female (VNMN), ‘Rog.246’, “[NW Vietnam:] Hoa Binh, Yen Thuy, fruit orchard, MT 20–30.x.2002, KD Long”; 1 female (VNMN), ‘Rog.567’, 1 male (VNMN), ‘Rog.569’, “[NE Vietnam:] Ha Giang, Vi Xuyen, Dao Duc, secondary forest, sweeping 20.x.2007, KD Long”.

##### Notes.

Female: body length 5.0-5.1 mm, length of fore wing 4.2 mm, antenna 6.3 mm; male: body length 4.0 mm, length of fore wing 3.5 mm, antenna 4.5 mm; in dorsal view occipital carina weakly concave (Fig. 32); basal length of second submarginal cell of fore wing 1.6 times as long as its apical width (16:10); vein M+CU of hind wing 1.3-1.6 times as long as vein 1-M (Fig. 34); vein 1-SC+R curved apically; vein 2-SC+R of hind wing subquadrate, swollen apically (Fig. 34); hind tarsal claw simple (Fig. 31); propodeum areolate medially (Fig. 33); ovipositor sheath 1.55 times as long as hind inner spur (14:9).

##### Distribution.

China; NW Vietnam: Hoa Binh (Yen Thuy), NE Vietnam: Ha Giang (Vi Xuyen).

## Supplementary Material

XML Treatment for Iporhogas

XML Treatment for Iporhogas albilateralis

XML Treatment for Iporhogas contrastus

XML Treatment for Iporhogas simulatus

XML Treatment for Iporhogas tricoloratus

XML Treatment for Iporhogas guangxiensis
